# Supplementary material for: Adolescent social isolation creates a latent vulnerability in maternal care with intergenerational social consequences, rescued by experienced mothers
Source: bioRxiv. 2026 Apr 16:2026.04.14.718476. Preprint. [Version 2] doi: 10.64898/2026.04.14.718476 (PMC13104813; doi:10.64898/2026.04.14.718476)
Supplement: 1 [file NIHPP2026.04.14.718476v2-supplement-1.pdf]

bioRxiv preprint doi: https://doi.org/10.64898/2026.04.14.718476; this version posted April 16, 2026. The copyright holder for this preprint (which was not certified by peer review) is the author/funder, who has granted bioRxiv a license to display the preprint in perpetuity. It is made available under aCC-BY 4.0 International license.

Supporting Information for

Adolescent social isolation creates a latent vulnerability in maternal care with intergenerational social consequences, rescued by experienced mothers

Jose Francis-Oliveira[a], Rinako Tanaka[a], Matthew Shen[a], Emily Cruvinel[a,b], Shin-ichi Kano[a,c], Minae Niwa[a,c,d,1.]

[a]Department of Psychiatry and Behavioral Neurobiology, University of Alabama at Birmingham School of Medicine, Birmingham, AL, USA

[b]Department of Physiology and Biophysics, Sao Paulo University, Sao Paulo, Brazil

[c]Department of Neurobiology, University of Alabama at Birmingham School of Medicine, Birmingham, AL, USA

[d]Department of Biomedical Engineering, University of Alabama at Birmingham School of Engineering, Birmingham, AL, USA

[1]To whom correspondence may be addressed.

Minae Niwa, Ph.D.

Email: mniwa@uabmc.edu

bioRxiv preprint doi: https://doi.org/10.64898/2026.04.14.718476; this version posted April 16, 2026. The copyright holder for this preprint (which was not certified by peer review) is the author/funder, who has granted bioRxiv a license to display the preprint in perpetuity. It is made available under aCC-BY 4.0 International license.

This PDF file includes:

Figures S1 to S6

Table S1

bioRxiv preprint doi: https://doi.org/10.64898/2026.04.14.718476; this version posted April 16, 2026. The copyright holder for this preprint (which was not certified by peer review) is the author/funder, who has granted bioRxiv a license to display the preprint in perpetuity. It is made available under aCC-BY 4.0 International license.

Supplementary Figure 1

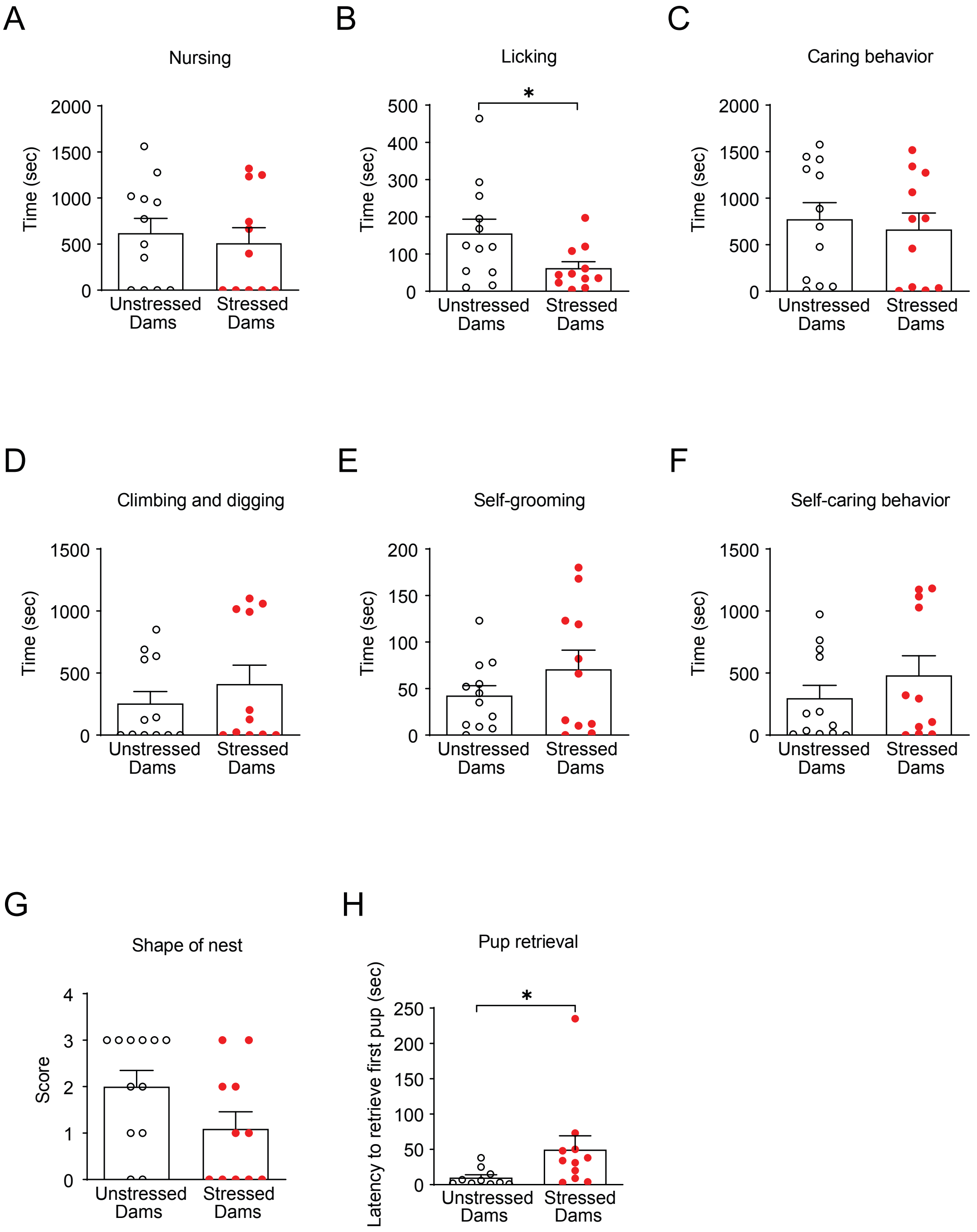

bioRxiv preprint doi: https://doi.org/10.64898/2026.04.14.718476; this version posted April 16, 2026. The copyright holder for this preprint (which was not certified by peer review) is the author/funder, who has granted bioRxiv a license to display the preprint in perpetuity. It is made available under aCC-BY 4.0 International license.

Supplemental Figure 1. Early postpartum maternal care deficits are limited at delivery. (A-C) Caring behavior toward pups shortly after delivery (postpartum day 1) was largely unchanged, except for pup licking. (D-F) Self-care behaviors, including climbing/digging and self-grooming, were not affected. (G) Nest scores were similar between unstressed and stressed dams. (H) Pup retrieval latency was increased in stressed dams, indicating reduced maternal responsiveness. Data are shown as means ± SEM, N = 11-12 per group. *$p < 0.05$. Statistical tests: Mann-Whitney U (A-D, F-H) and Welch's t-tests (E) were applied as appropriate. Detailed statistics are provided in **Supplementary Table 1**.

bioRxiv preprint doi: https://doi.org/10.64898/2026.04.14.718476; this version posted April 16, 2026. The copyright holder for this preprint (which was not certified by peer review) is the author/funder, who has granted bioRxiv a license to display the preprint in perpetuity. It is made available under aCC-BY 4.0 International license.

Supplementary Figure 2

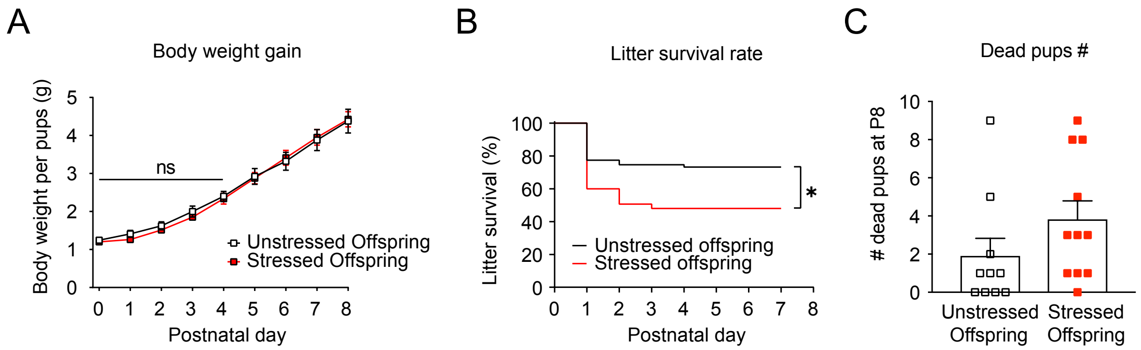

bioRxiv preprint doi: https://doi.org/10.64898/2026.04.14.718476; this version posted April 16, 2026. The copyright holder for this preprint (which was not certified by peer review) is the author/funder, who has granted bioRxiv a license to display the preprint in perpetuity. It is made available under aCC-BY 4.0 International license.

Supplemental Figure 2. Effects of impaired maternal caregiving on offspring growth and survival. (A) Pup body weight gain over the first postnatal week was similar between groups. (B) Survival rates were reduced in stressed offspring compared to unstressed controls. (C) Number of dead pups per litter was not significantly different, although stressed litters tended to have more deaths. Data are shown as means ± SEM, N = 10-11 litters per group. $^{*}p < 0.05$. Statistical tests: Mann-Whitney U and Student's t Test (A, C) and Log-rank Mantel-Cox (B) were applied as appropriate. Detailed statistics are provided in **Supplementary Table 1**.

bioRxiv preprint doi: https://doi.org/10.64898/2026.04.14.718476; this version posted April 16, 2026. The copyright holder for this preprint (which was not certified by peer review) is the author/funder, who has granted bioRxiv a license to display the preprint in perpetuity. It is made available under aCC-BY 4.0 International license.

Supplementary Figure 3

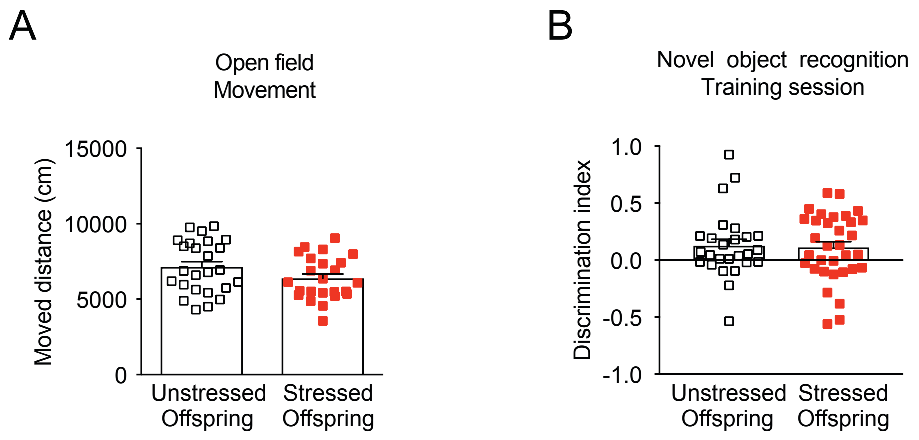

bioRxiv preprint doi: https://doi.org/10.64898/2026.04.14.718476; this version posted April 16, 2026. The copyright holder for this preprint (which was not certified by peer review) is the author/funder, who has granted bioRxiv a license to display the preprint in perpetuity. It is made available under aCC-BY 4.0 International license.

Supplemental Figure 3. Validation of behavioral measures. (A) Total distance moved in the open field test was similar between groups, indicating no differences in locomotor activity. (B) Discrimination index in the novel object recognition test during the training session was similar across groups. Data are shown as means ± SEM, N = 24-25 per group. *$p < 0.05$. Statistical tests: Student's t-test (A) and Mann-Whitney U test (B) were applied as appropriate. Detailed statistics are provided in **Supplementary Table 1**.

bioRxiv preprint doi: https://doi.org/10.64898/2026.04.14.718476; this version posted April 16, 2026. The copyright holder for this preprint (which was not certified by peer review) is the author/funder, who has granted bioRxiv a license to display the preprint in perpetuity. It is made available under aCC-BY 4.0 International license.

Supplementary Figure 4

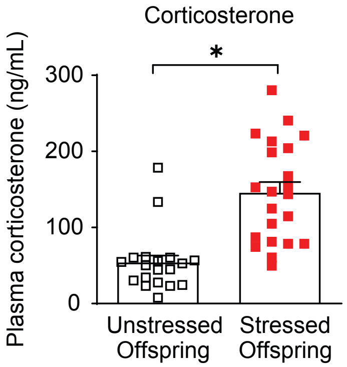

bioRxiv preprint doi: https://doi.org/10.64898/2026.04.14.718476; this version posted April 16, 2026. The copyright holder for this preprint (which was not certified by peer review) is the author/funder, who has granted bioRxiv a license to display the preprint in perpetuity. It is made available under aCC-BY 4.0 International license.

Supplemental Figure 4. Increased plasma corticosterone levels in stressed offspring. Plasma corticosterone levels were elevated in adult offspring reared by stressed dams. Data are shown as means ± SEM, N = 19-22 per group. *$p < 0.05$. Statistical tests: Mann-Whitney U test was applied. Detailed statistics are provided in **Supplementary Table 1**.

bioRxiv preprint doi: https://doi.org/10.64898/2026.04.14.718476; this version posted April 16, 2026. The copyright holder for this preprint (which was not certified by peer review) is the author/funder, who has granted bioRxiv a license to display the preprint in perpetuity. It is made available under aCC-BY 4.0 International license.

Supplementary Figure 5

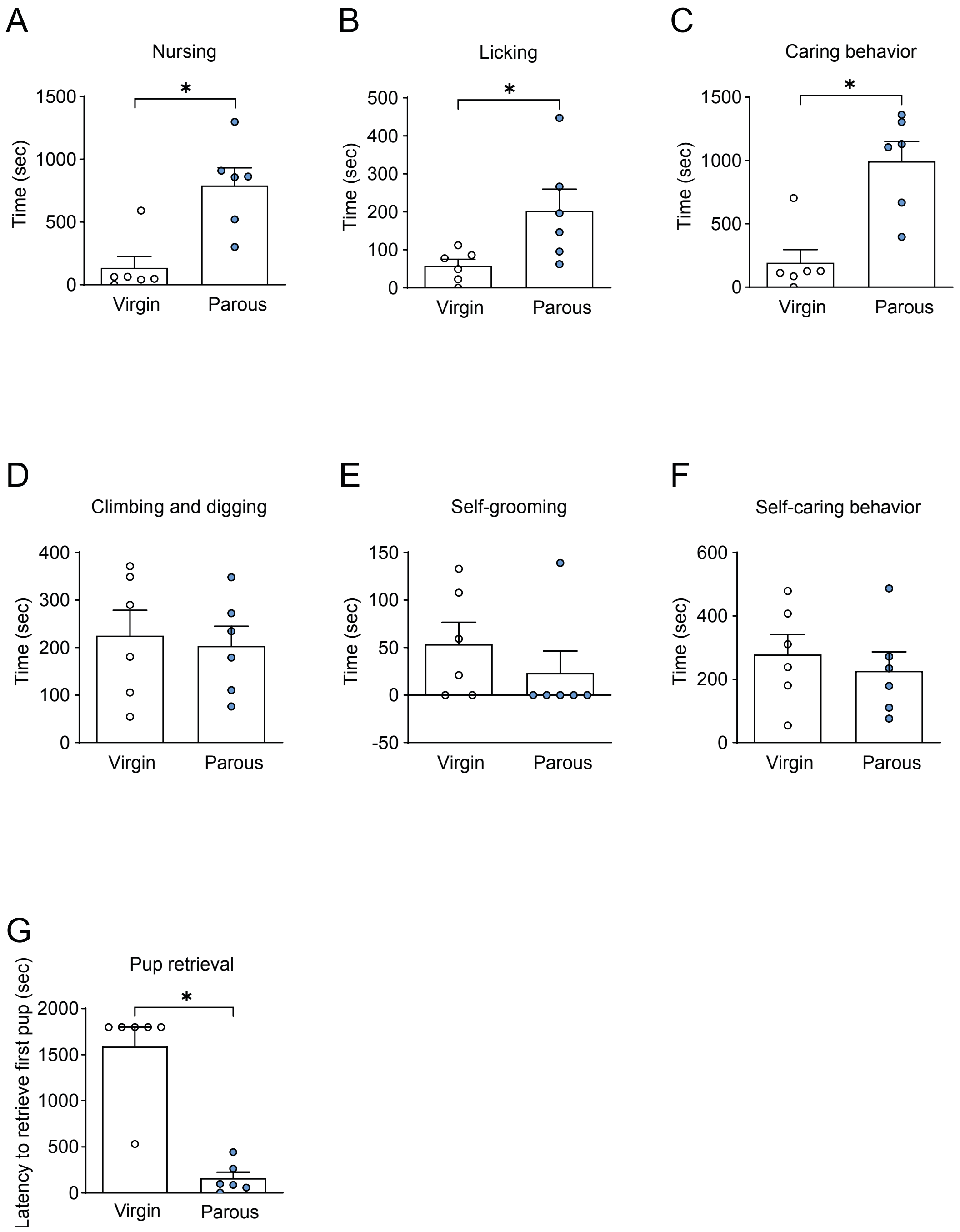

bioRxiv preprint doi: https://doi.org/10.64898/2026.04.14.718476; this version posted April 16, 2026. The copyright holder for this preprint (which was not certified by peer review) is the author/funder, who has granted bioRxiv a license to display the preprint in perpetuity. It is made available under aCC-BY 4.0 International license.

Supplemental Figure 5. Parous mice display robust maternal behavior. (A-C) Pup-directed behaviors, including nursing, licking, and retrieval, were increased in parous females compared to virgins. (D-F) Self-care behaviors were similar between virgin and parous females. (G) Pup retrieval latency was reduced in parous females, reflecting enhanced maternal responsiveness. Data are shown as means ± SEM, N = 6 per group. *$p < 0.05$. Statistical tests: Mann-Whitney U test (A, C, E, G) and Student's t-test (B, D, F) were applied as appropriate. Detailed statistics are provided in **Supplementary Table 1**.

bioRxiv preprint doi: https://doi.org/10.64898/2026.04.14.718476; this version posted April 16, 2026. The copyright holder for this preprint (which was not certified by peer review) is the author/funder, who has granted bioRxiv a license to display the preprint in perpetuity. It is made available under aCC-BY 4.0 International license.

Supplementary Figure 6

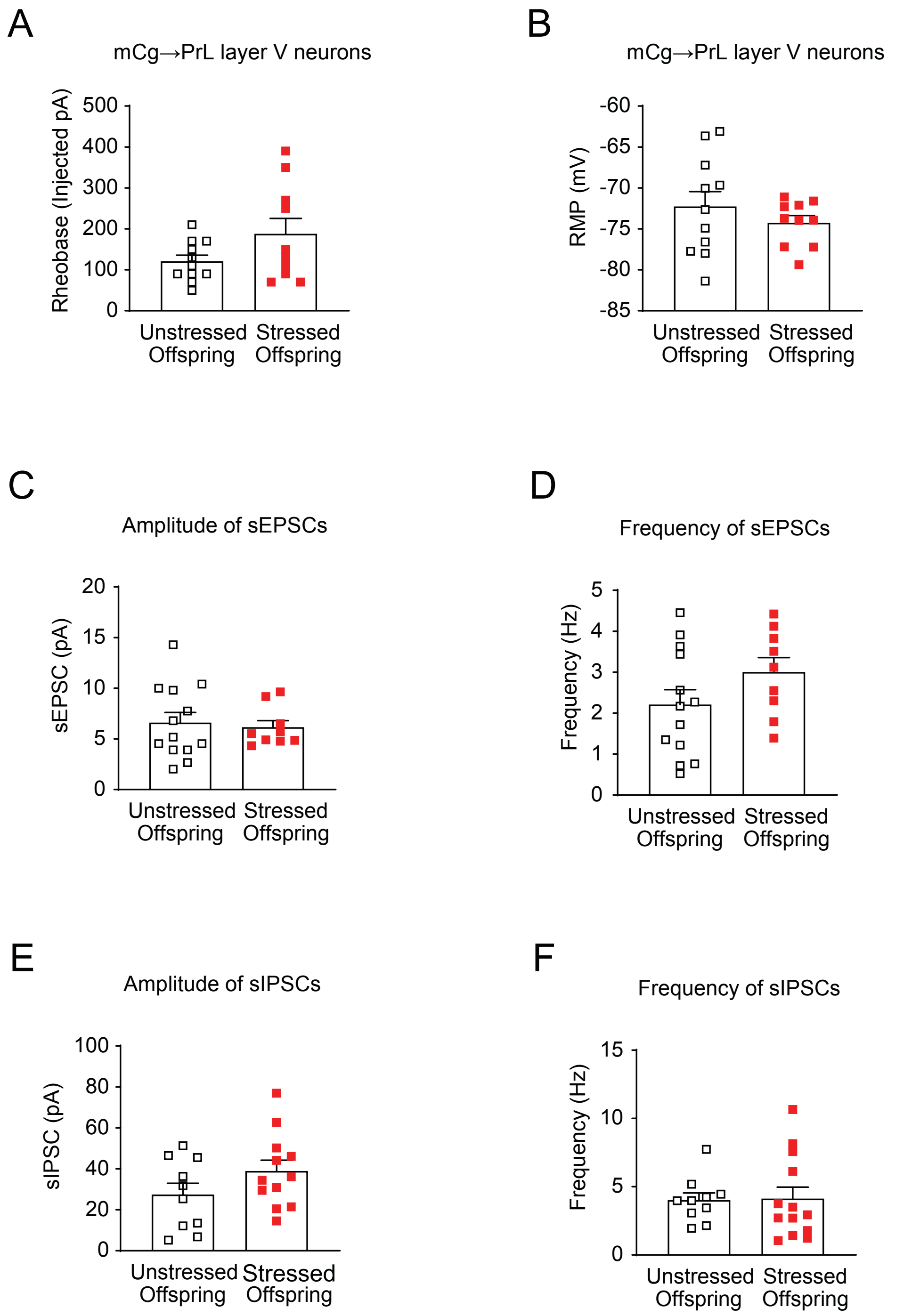

bioRxiv preprint doi: https://doi.org/10.64898/2026.04.14.718476; this version posted April 16, 2026. The copyright holder for this preprint (which was not certified by peer review) is the author/funder, who has granted bioRxiv a license to display the preprint in perpetuity. It is made available under aCC-BY 4.0 International license.

Supplemental Figure 6. Electrophysiological characterization of mCg→PrL neurons in stressed offspring. (A) Rheobase of these neurons showed no change. (B) Resting membrane potential was similar between groups. (C-F) Amplitude and frequency of spontaneous excitatory and inhibitory currents were unchanged, indicating intact presynaptic input. Data are shown as means ± SEM, N = 10-12 cells per group. Each group included cells obtained from at least three animals. Statistical tests: Two-way mixed ANOVA (A) and Student's/Welch's t-tests (B-G) were applied. Detailed statistics are provided in **Supplementary Table 1**.

bioRxiv preprint doi: https://doi.org/10.64898/2026.04.14.718476; this version posted April 16, 2026. The copyright holder for this preprint (which was not certified by peer review) is the author/funder, who has granted bioRxiv a license to display the preprint in perpetuity. It is made available under aCC-BY 4.0 International license.

Supplementary Table 1. Statistical analyses for all results. For each dataset, sample sizes, normality tests, statistical tests performed, *p* values, effect sizes, and post hoc comparisons are reported. For repeated-measures ANOVAs, selected within-group factors were included in post hoc analyses.

Figure 1	**Shapiro-Wilk test**	**Levene's test**	
A	Nursing		**Student's t-Test Independent samples, 2-tailed**
	Unstressed *p* = 0.111	*p* = 0.196	*t* (21) = 2.179 , *p* = 0.041
	Stressed *p* = 0.090		Cohen's d = 0.909
B	Licking		**Welch t-Test Independent samples, 2-tailed**
	Unstressed *p* = 0.388	*p* = 0.006	*t* (16) = 2.176 , *p* = 0.045
	Stressed *p* = 0.291		Cohen's d = 0.883
C	Caring Behavior		**Student's t-Test Independent samples, 2-tailed**
	Unstressed *p* = 0.109	*p* = 0.202	*t* (21) = 2.506 , *p* = 0.021
	Stressed *p* = 0.133		Cohen's d = 1.046
D	Climbing and Digging		**Mann Whitney U test**
	Unstressed *p* <0.001		Unstressed x Stressed *p* = 0.069 *U* = 96.00
	Stressed *p* = 0.031		*r* = 0.464 *z* = 1.86
E	Self-grooming		**Mann Whitney U test**
	Unstressed *p* = 0.199		Unstressed x Stressed *p* = 0.740 *U* = 71.50
	Stressed *p* = 0.020		*r* = 0.0848 *z* = 0.34
F	Self-caring Behavior		**Mann Whitney U test**
	Unstressed *p* = 0.030		Unstressed x Stressed *p* = 0.104 *U* = 93.00
	Stressed *p* = 0.084		*r* = 0.4158 *z* = 1.66
G	Shape of Nest		**Mann Whitney U test**
	Unstressed *p* = 0.001		Unstressed x Stressed *p* = 0.019 *U* = 28.00
	Stressed *p* = 0.150		*r* = 0.6185 *z* = 2.47
H	Pup Retrieval 1st Pup		**Mann Whitney U test**
	Unstressed *p* <0.001		Unstressed x Stressed *p* = 0.019 *U* = 96.00
	Stressed *p* = 0.012		*r* = 0.5835 *z* = 2.33
	**Sample Size**		
	Unstressed dams *n* = 12		
	Stressed dams *n* = 11		
**Figure 2**	**Shapiro-Wilk test**	**Levene's test**	
A	Sociability		**Welch's t-Test Independent samples, 2-tailed**
	Unstressed Offspring *p* = 0.643	*p* = 0.013	*t* (40) = 2.338 , *p* = 0.013
	Stressed Offspring *p* = 0.312		Glass's Delta = 0.576
B	Social Novelty		**Welch's t-Test Independent samples, 2-tailed**
	Unstressed Offspring *p* = 0.906	*p* = 0.006	*t* (40) = 2.446 , *p* = 0.020
	Stressed Offspring *p* = 0.202		Glass's Delta = 0.422
C	Social Olfactory Recognition		**Two-Way mixed ANOVA**
	Unstressed group *p* = 0.875	*p* <0.05	Interaction F(6,132) = 3.013 , *p* = 0.024 , partial η^2 = 0.120
	Stressed group *p* = 0.590		Scent (W) F(1,22) = 9.778 , *p* <0.001 , partial η^2 = 0.308
	Water *p* = 0.009		Stress (B) F(1,22) = 5.742 , *p* = 0.026 , partial η^2 = 0.207
	Familiar 1 *p* = 0.007		
	Stranger 1 *p* = 0.004		post hoc (Bonferroni)
			Familiar 1 Unstressed x Stressed *p* = 0.037
			Stranger 1 Unstressed x Stressed *p* <0.001
D	Novel Object Recognition		**Mann Whitney U test**
	Unstressed Offspring *p* = 0.019	*p* = 0.116	Unstressed x Stressed *p* = 0.368 *U* = 370.0
	Stressed Offspring *p* = 0.369		*r* = 0.225 *z* = 0.90
E	Open Field Test		**Student's t-Test Independent samples, 2-tailed**
	Unstressed Offspring *p* = 0.248	*p* = 0.782	*t* (47) = 0.384 , *p* = 0.702
	Stressed Offspring *p* = 0.107		Glass's Delta = 0.112
F	Elevated Plus Maze		**Student's t-Test Independent samples, 2-tailed**
	Unstressed Offspring *p* = 0.598	*p* = 0.185	*t* (47) = 1.946 , *p* = 0.058
	Stressed Offspring *p* = 0.281		Glass's Delta = 0.624
G	Light/Dark Box		**Mann Whitney U test**
	Unstressed Offspring *p* = 0.028		Unstressed x Stressed *p* = 0.810 *U* = 49.00
	Stressed Offspring *p* = 0.964		*r* = 0.06 *z* = 0.24
	**Sample Size**		
	Unstressed Offspring *n* = 11~19		
	Stressed Offspring *n* = 13~23		
**Figure 3**	**Shapiro-Wilk test**	**Levene's test**	
B	Sociability		**Two-Way ANOVA**

bioRxiv preprint doi: https://doi.org/10.64898/2026.04.14.718476; this version posted April 16, 2026. The copyright holder for this preprint (which was not certified by peer review) is the author/funder, who has granted bioRxiv a license to display the preprint in perpetuity. It is made available under aCC-BY 4.0 International license.

	Unstressed Vehicle p =	0.197	p = 0.056	Interaction	F(3,24) = 6.820 ,	p = 0.017 ,	partial η^2 = 0.254
	Unstressed CNO p =	0.282		Stress (B)	F(1,24) = 2.910 ,	p = 0.104 ,	partial η^2 = 0.127
	Stressed Vehicle p =	0.282		CNO (B)	F(1,24) = 4.435 ,	p = 0.048 ,	partial η^2 = 0.182
	Stressed CNO p =	0.649					
				post hoc (Bonferroni)			
				Unstressed	Vehicle x CNO	p = 0.725	
				Stressed	Vehicle x CNO	p = 0.003	
				Vehicle	Unstressed x Stressed	p = 0.006	
				CNO	Unstressed x Stressed	p = 0.529	
**C**	Social Novelty			**Two-Way ANOVA**			
	Unstressed Vehicle p =	0.817	p = 0.047	Interaction	F(3,24) = 9.256 ,	p = 0.006 ,	partial η^2 = 0.316
	Unstressed CNO p =	0.486		Stress (B)	F(1,24) = 1.495 ,	p = 0.236 ,	partial η^2 = 0.070
	Stressed Vehicle p =	0.620		CNO (B)	F(1,24) = 6.823 ,	p = 0.017 ,	partial η^2 = 0.254
	Stressed CNO p =	0.797					
				post hoc (Bonferroni)			
				Unstressed	Vehicle x CNO	p = 0.764	
				Stressed	Vehicle x CNO	p = 0.001	
				Vehicle	Unstressed x Stressed	p = 0.007	
				CNO	Unstressed x Stressed	p = 0.213	
**E**	Sociability			**Student's t-Test Independent samples, 2-tailed**			
	Unstressed Vehicle p =	0.304	p = 0.136	t (10) = 3.115 ,	p = 0.011		
	Unstressed CNO p =	0.215		Hedge's g = 1.660			
**F**	Social Novelty			**Student's t-Test Independent samples, 2-tailed**			
	Unstressed Vehicle p =	0.580	p = 0.589	t (10) = 2.763 ,	p = 0.020		
	Unstressed CNO p =	0.849		Hedge's g = 1.472			
	**Sample Size**						
	Unstressed Vehicle n =	6					
	Unstressed CNO n =	6					
	Stressed Vehicle n =	6					
	Stressed CNO n =	6					
**Figure 4**	**Shapiro-Wilk test**		**Levene's test**				
**B**	Nursing			**Mann Whitney U test**			
	Virgin Unstressed p =	0.017		Virgin	Unstressed x Stressed	p = 0.012	U = 29.50
	Parous Unstressed p =	0.210				r = 0.708	z = 2.45
	Virgin Stressed p =	0.120		Parous	Unstressed x Stressed	p = 0.078	U = 41.50
	Parous Stressed p	<0.001				r = 0.5084	z = 1.76
				Stressed	Virgin x Parous	p = 0.004	U = 121.00
						r = 0.8167	z = 2.83
				Unstressed	Virgin x Parous	p = 0.514	U = 83.50
						r = 0.192	z = 0.66
**C**	Licking			**Mann Whitney U test**			
	Virgin Unstressed p =	0.016		Virgin	Unstressed x Stressed	p = 0.002	U = 21.00
	Parous Unstressed p =	0.027				r = 0.850	z = 2.95
	Virgin Stressed p =	0.028		Parous	Unstressed x Stressed	p = 0.887	U = 75.00
	Parous Stressed p =	0.003				r = 0.050	z = 0.17
				Stressed	Virgin x Parous	p = 0.003	U = 121.50
						r = 0.825	z = 2.86
				Unstressed	Virgin x Parous	p = 0.713	U = 65.00
						r = 0.117	z = 0.40
**D**	Caring Behavior			**Mann Whitney U test**			
	Virgin Unstressed p =	0.045		Virgin	Unstressed x Stressed	p = 0.001	U = 18.00
	Parous Unstressed p =	0.596				r = 0.900	z = 3.12
	Virgin Stressed p =	0.047		Parous	Unstressed x Stressed	p = 0.114	U = 44.00
	Parous Stressed p =	0.001				r = 0.467	z = 1.62
				Stressed	Virgin x Parous	p = 0.001	U = 127.00
						r = 0.917	z = 3.18
				Unstressed	Virgin x Parous	p = 0.630	U = 81.00
						r = 0.150	z = 0.52
**E**	Climbing and Digging			**Mann Whitney U test**			
	Virgin Unstressed p =	0.122		Virgin	Unstressed x Stressed	p = 0.001	U = 126.00
	Parous Unstressed p =	0.012				r = 0.900	z = 3.12
	Virgin Stressed p =	0.351		Parous	Unstressed x Stressed	p = 0.198	U = 94.50
	Parous Stressed p =	0.022				r = 0.375	z = 1.30
				Stressed	Virgin x Parous	p = 0.198	U = 49.00
						r = 0.383	z = 1.33
				Unstressed	Virgin x Parous	p = 0.551	U = 83.00
						r = 0.183	z = 0.64
**F**	Self-grooming			**Mann Whitney U test**			
	Virgin Unstressed p	<0.001		Virgin	Unstressed x Stressed	p = 0.671	U = 79.50
	Parous Unstressed p	<0.001				r = 0.149	z = 0.52
	Virgin Stressed p	<0.001		Parous	Unstressed x Stressed	p = 0.128	U = 45.50

bioRxiv preprint doi: https://doi.org/10.64898/2026.04.14.718476; this version posted April 16, 2026. The copyright holder for this preprint (which was not certified by peer review) is the author/funder, who has granted bioRxiv a license to display the preprint in perpetuity. It is made available under aCC-BY 4.0 International license.

	Parous Stressed *p*	<0.001				*r* =	0.581	*z* =	2.01
				Stressed	Virgin x Parous	*p* =	0.128	*U* =	45.50
						r =	0.581	*z* =	2.01
				Unstressed	Virgin x Parous	*p* =	0.443	*U* =	85.50
						r =	0.268	*z* =	0.93
**G**	Self-caring Behavior				**Mann Whitney U test**				
	Virgin Unstressed *p* =	0.179		Virgin	Unstressed x Stressed	*p* =	0.002	*U* =	123.00
	Parous Unstressed *p* =	0.173				*r* =	0.850	*z* =	2.94
	Virgin Stressed *p* =	0.604		Parous	Unstressed x Stressed	*p* =	0.319	*U* =	89.50
	Parous Stressed *p* =	0.023				*r* =	0.292	*z* =	1.01
				Stressed	Virgin x Parous	*p* =	0.128	*U* =	45.00
						r =	0.450	*z* =	1.56
				Unstressed	Virgin x Parous	*p* =	0.514	*U* =	84.00
						r =	0.200	*z* =	0.69
**H**	Shape of Nest				**Mann Whitney U test**				
	Virgin Unstressed *p*	<0.001		Virgin	Unstressed x Stressed	*p* =	0.020	*U* =	32.00
	Parous Unstressed *p*	<0.001				*r* =	0.719	*z* =	2.49
	Virgin Stressed *p* =	0.010		Parous	Unstressed x Stressed	*p* =	0.319	*U* =	90.00
	Parous Stressed *p*	<0.001				*r* =	0.357	*z* =	1.24
				Stressed	Virgin x Parous	*p* =	0.010	*U* =	115.50
						r =	0.790	*z* =	2.74
				Unstressed	Virgin x Parous	*p* =	0.514	*U* =	60.00
						r =	0.234	*z* =	0.81
**I**	Pup Retrieval 1st Pup				**Mann Whitney U test**				
	Virgin Unstressed *p*	<0.001		Virgin	Unstressed x Stressed	*p*	<0.001	*U* =	137.00
	Parous Unstressed *p* =	0.001				*r* =	1.085	*z* =	3.76
	Virgin Stressed *p* =	0.272		Parous	Unstressed x Stressed	*p* =	0.977	*U* =	71.50
	Parous Stressed *p* =	0.002				*r* =	0.008	*z* =	0.03
				Stressed	Virgin x Parous	*p*	<0.001	*U* =	10.00
						r =	1.034	*z* =	3.58
				Unstressed	Virgin x Parous	*p* =	0.410	*U* =	86.50
						r =	0.242	*z* =	0.84
	**Sample Size**								
	Virgin Unstressed *n* =	12							
	Parous Unstressed *n* =	12							
	Virgin Stressed *n* =	12							
	Parous Stressed *n* =	12							
**Figure 5**	**Shapiro-Wilk test**		**Levene's test**						
**A**	Sociability				**Kruskal-Wallis**				
	Unstressed *p* =	0.049		Stress (B)	*H* (2) = 8.180 ,	*p* =	0.017		
	Stressed *p* =	0.229							
	Parous *p* =	0.236			post hoc (Bonferroni)				
				Virgin	Unstressed x Stressed	*p* =	0.008		
				Virgin	Unstressed x Parous	*p* =	0.969		
				Virgin	Stressed x Parous	*p* =	0.041		
**B**	Social Novelty				**Welch's One-Way ANOVA**				
	Unstressed *p* =	0.480	*p* = 0.006	Stress (B)	F (2,38) = 8.470 ,	*p*	<0.001		
	Stressed *p* =	0.080							
	Parous *p* =	0.971			post hoc (Bonferroni)				
				Virgin	Unstressed x Stressed	*p* =	0.002		
				Virgin	Unstressed x Parous	*p* =	1.000		
				Virgin	Stressed x Parous	*p* =	0.010		
	**Sample Size**								
	Virgin Unstressed Offspring *n* =	18							
	Virgin Stressed Offspring *n* =	16							
	Parous Stressed Offspring *n* =	7							
**Figure 6**									
**E**	Firing Frequency mCg→PrL				**Two-Way mixed ANOVA**				
	Unstressed Offspring *p* =	0.711	*p* >0.05	Interaction	F(54,837) = 3.382 ,	*p* =	0.021 ,	partial η^2 =	0.179
	Stressed Offspring *p* =	0.543		Current (W)	F(27,837) = 150.042 ,	*p*	<0.001 ,	partial η^2 =	0.829
	Parous Offspring *p* =	0.694		Stress (B)	F(1,31) = 4.499 ,	*p* =	0.019 ,	partial η^2 =	0.225
					post hoc (Bonferroni)				
					Stressed x Unstressed	*p*	<0.001		
					Stressed x Parous	*p*	<0.001		
					Unstressed x Parous	*p* =	0.999		
	**Sample Size**								
	Virgin Unstressed Offspring *n* =	11 cells							
	Virgin Stressed Offspring *n* =	10 cells							
	Parous Stressed Offspring *n* =	13 cells							
**Supplementary**	**Shapiro-Wilk test**		**Levene's test**						

bioRxiv preprint doi: https://doi.org/10.64898/2026.04.14.718476; this version posted April 16, 2026. The copyright holder for this preprint (which was not certified by peer review) is the author/funder, who has granted bioRxiv a license to display the preprint in perpetuity. It is made available under aCC-BY 4.0 International license.

Figure	Measure	Shapiro-Wilk	Levene's test	Test / Comparison	Statistic	p / r	U / z
**Figure 1**							
**A**	Nursing			**Mann Whitney U test**			
	Unstressed *p* =	0.147		Unstressed x Stressed		*p* = 0.651	*U* = 58.00
	Stressed *p* =	0.011				*r* = 0.127	*z* = 0.51
**B**	Licking			**Mann Whitney U test**			
	Unstressed *p* =	0.174		Unstressed x Stressed		*p* = 0.044	*U* = 33.00
	Stressed *p* =	0.045				*r* = 0.5078	*z* = 2.03
**C**	Caring Behavior			**Mann Whitney U test**			
	Unstressed *p* =	0.065		Unstressed x Stressed		*p* = 0.260	*U* = 47.00
	Stressed *p* =	0.029				*r* = 0.2923	*z* = 1.17
**D**	Climbing and Digging			**Mann Whitney U test**			
	Unstressed *p* =	0.002		Unstressed x Stressed		*p* = 0.347	*U* = 82.00
	Stressed *p* =	0.001				*r* = 0.2515	*z* = 1.01
**E**	Self-grooming			**Welch t-Test Independent samples, 2-tailed**			
	Unstressed *p* =	0.288	*p* = 0.015	*t* (15)	= 1.221	*p* = 0.241	
	Stressed *p* =	0.082		Cohen's d	= 0.523		
**F**	Self-caring Behavior			**Mann Whitney U test**			
	Unstressed *p* =	0.007		Unstressed x Stressed		*p* = 0.413	*U* = 80.00
	Stressed *p* =	0.004				*r* = 0.2155	*z* = 0.86
**G**	Shape of Nest			**Mann Whitney U test**			
	Unstressed *p* =	0.007		Unstressed x Stressed		*p* = 0.104	*U* = 39.00
	Stressed *p* =	0.014				*r* = 0.433	*z* = 1.73
**H**	Pup Retrieval 1st Pup			**Mann Whitney U test**			
	Unstressed *p* =	0.005					
	Stressed *p*	<0.001		Unstressed x Stressed		*p* = 0.008	*U* = 92.00
						r = 0.6523	*z* = 2.61
	**Sample Size**						
	Unstressed dams *n* =	12					
	Stressed dams *n* =	11					
**Supplementary Figure 2**	**Shapiro-Wilk test**		**Levene's test**				
**A**	Pups Body Weight			**Mann Whitney U test**			
	P0 Unstressed *p* =	0.195		P0 Unstressed x Stressed		*p* = 0.481	*U* = 28.50
	Stressed *p* =	0.005				*r* = 0.197	*z* = 0.79
				**Mann Whitney U test**			
	P1 Unstressed *p* =	0.239		P1 Unstressed x Stressed		*p* = 0.277	*U* = 24.50
	Stressed *p* =	0.024				*r* = 0.287	*z* = 1.15
				**Student's t-Test Independent samples, 2-tailed**			
	P2 Unstressed *p* =	0.202	*p* = 0.398	P2 *t* (15)	= 0.848	*p* = 0.410	
	Stressed *p* =	0.343		Cohen's d	= 0.412		
				**Mann Whitney U test**			
	P3 Unstressed *p* =	0.040		P3 Unstressed x Stressed		*p* = 0.606	*U* = 30.00
	Stressed *p* =	0.408				*r* = 0.147	*z* = 0.59
				**Student's t-Test Independent samples, 2-tailed**			
	P4 Unstressed *p* =	0.297	*p* = 0.989	P4 *t* (15)	= 0.330	*p* = 0.746	
	Stressed *p* =	0.619		Cohen's d	= 0.160		
**B**				**Log-rank Mantel-Cox test**			
				χ^2 (1)	= 9.501	*p* = 0.002	
**C**	# Dead pups at P8			**Mann Whitney U test**			
	Unstressed litter *p* =	0.001		Unstressed x Stressed		*p* = 0.099	*U* = 78.50
	Stressed litter *p* =	0.087				*r* = 0.422	*z* = 1.69
	**Sample Size**						
	Unstressed Litters *n* =	10					
	Stressed Litters *n* =	11					
	Total Unstressed Pups *n* =	71					
	Total Stressed Pups *n* =	75					
**Supplementary Figure 3**	**Shapiro-Wilk test**		**Levene's test**				
**A**	Open Field			**Student's t-Test Independent samples, 2-tailed**			
	Unstressed Offspring *p* =	0.133	*p* = 0.139	*t* (47)	= 1.685	*p* = 0.099	
	Stressed Offspring *p* =	0.405		Glass's Delta	= 0.539		
**B**	Novelty Object Recognition			**Mann Whitney U test**			
	Unstressed Offspring *p* =	0.010		Unstressed x Stressed		*p* = 0.831	*U* = 443.00
	Stressed Offspring *p* =	0.556				*r* = 0.054	*z* = 0.21
	**Sample Size**						
	Unstressed Offspring *n* =	25~26					

bioRxiv preprint doi: https://doi.org/10.64898/2026.04.14.718476; this version posted April 16, 2026. The copyright holder for this preprint (which was not certified by peer review) is the author/funder, who has granted bioRxiv a license to display the preprint in perpetuity. It is made available under aCC-BY 4.0 International license.

	Stressed Offspring *n* = 24~33		
**Supplementary Figure 4**	**Shapiro-Wilk test**<br>Unstressed *p* <0.001<br>Stressed *p* = 0.305<br>**Sample Size**<br>Unstressed Offspring *n* = 19<br>Stressed Offspring *n* = 22	**Levene's test**	**Mann Whitney U test**<br>Unstressed x Stressed *p* <0.001 *U* = 384.0<br>*r* = 1.144 *z* = 4.58
**Supplementary Figure 5**<br>**A**	**Shapiro-Wilk test**<br>Nursing<br>Unstressed *p* <0.001<br>Stressed *p* = 0.730	**Levene's test**	**Mann Whitney U test**<br>Unstressed x Stressed *p* = 0.009 *U* = 34.00<br>*r* = 0.641 *z* = 2.56
**B**	Licking<br>Unstressed *p* = 0.896<br>Stressed *p* = 0.473	*p* = 0.076	**Student's t-Test Independent samples, 2-tailed**<br>*t* (10) = 2.420 , *p* = 0.036<br>Cohen's d = 1.397
**C**	Caring Behavior<br>Unstressed *p* = 0.003<br>Stressed *p* = 0.291		**Mann Whitney U test**<br>Unstressed x Stressed *p* = 0.009 *U* = 34.00<br>*r* = 0.643 *z* = 2.57
**D**	Cimbing and Digging<br>Unstressed *p* = 0.503<br>Stressed *p* = 0.916	*p* = 0.312	**Student's t-Test Independent samples, 2-tailed**<br>*t* (10) = 0.319 , *p* = 0.756<br>Cohen's d = 0.184
**E**	Self-grooming<br>Unstressed *p* = 0.283<br>Stressed *p* <0.001		**Mann Whitney U test**<br>Unstressed x Stressed *p* = 0.310 *U* = 11.00<br>*r* = 0.313 *z* = 1.25
**F**	Self-caring<br>Unstressed *p* = 0.979<br>Stressed *p* = 0.455	*p* = 0.755	**Student's t-Test Independent samples, 2-tailed**<br>*t* (10) = 0.597 , *p* = 0.564<br>Cohen's d = 0.345
**G**	Pup Retrieval 1st Pup<br>Virgin *p* <0.001<br>Parous *p* = 0.199<br>**Sample Size**<br>Virgin *n* = 6<br>Parous *n* = 6		**Mann Whitney U test**<br>Unstressed x Stressed *p* = 0.002 *U* = 0.00<br>*r* = 0.745 *z* = 2.98
**Supplementary Figure 6**<br>**A**	**Shapiro-Wilk test**<br>Rheobase<br>Unstressed Offspring *p* = 0.159<br>Stressed Offspring *p* = 0.161	**Levene's test**<br>*p* = 0.002	**Welch's t-Test Independent samples, 2-tailed**<br>*t* (19) = 1.667 , *p* = 0.122<br>Glass's Delta = 0.567
**B**	Resting Membrane Potential<br>Unstressed Offspring *p* = 0.978<br>Stressed Offspring *p* = 0.222	*p* = 0.015	**Welch's t-Test Independent samples, 2-tailed**<br>*t* (19) = 0.978 , *p* = 0.345<br>Glass's Delta = 0.712
**C**	sEPSCs Amplitude<br>Unstressed Offspring *p* = 0.483<br>Stressed Offspring *p* = 0.028	*p* = 0.048	**Welch's t-Test Independent samples, 2-tailed**<br>*t* (20) = 0.369 , *p* = 0.716<br>Glass's Delta = 0.227
**D**	sEPSCs Frequency<br>Unstressed Offspring *p* = 0.203<br>Stressed Offspring *p* = 0.836	*p* = 0.476	**Student's t-Test Independent samples, 2-tailed**<br>*t* (20) = 1.501 , *p* = 0.149<br>Cohen's d = 0.651
**E**	sIPSCs Amplitude<br>Unstressed Offspring *p* = 0.080<br>Stressed Offspring *p* = 0.704	*p* = 0.873	**Student's t-Test Independent samples, 2-tailed**<br>*t* (20) = 1.509 , *p* = 0.147<br>Cohen's d = 0.646
**F**	sIPSCs Frequency<br>Unstressed Offspring *p* = 0.150<br>Stressed Offspring *p* = 0.275<br>**Sample Size**<br>Unstressed Offspring *n* = 11 cells<br>Stressed Offspring *n* = 10 cells	*p* = 0.038	**Welch's t-Test Independent samples, 2-tailed**<br>*t* (21) = 0.104 , *p* = 0.919<br>Glass's Delta = 0.034
